# Supplementary figures and images for: NP and 9311 are excellent population parents for screening QTLs of potassium-efficient rice
Source: PLoS One. 2023 Apr 14;18(4):e0284510. doi: 10.1371/journal.pone.0284510 (PMC10104323; doi:10.1371/journal.pone.0284510)

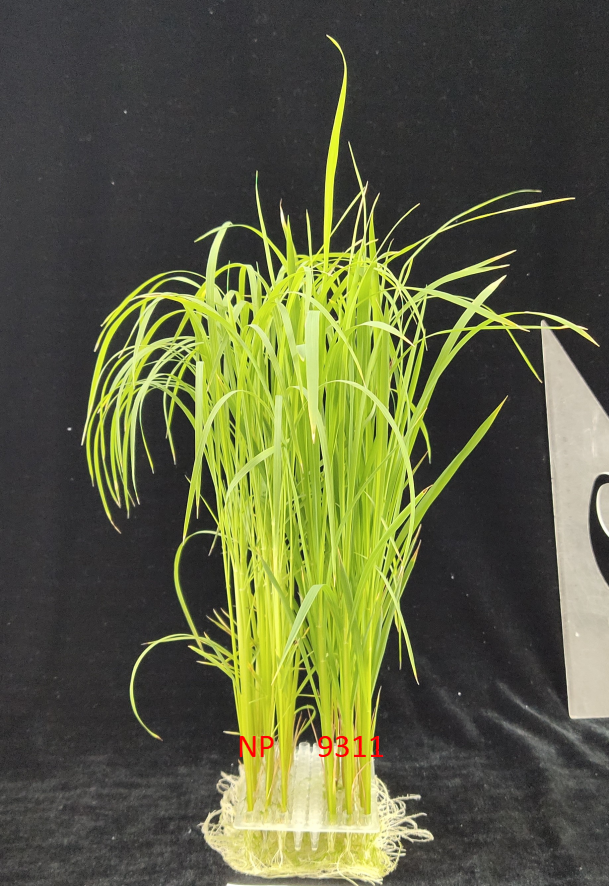

Supplement: S1 Fig — (TIF) [file pone.0284510.s001.tif]

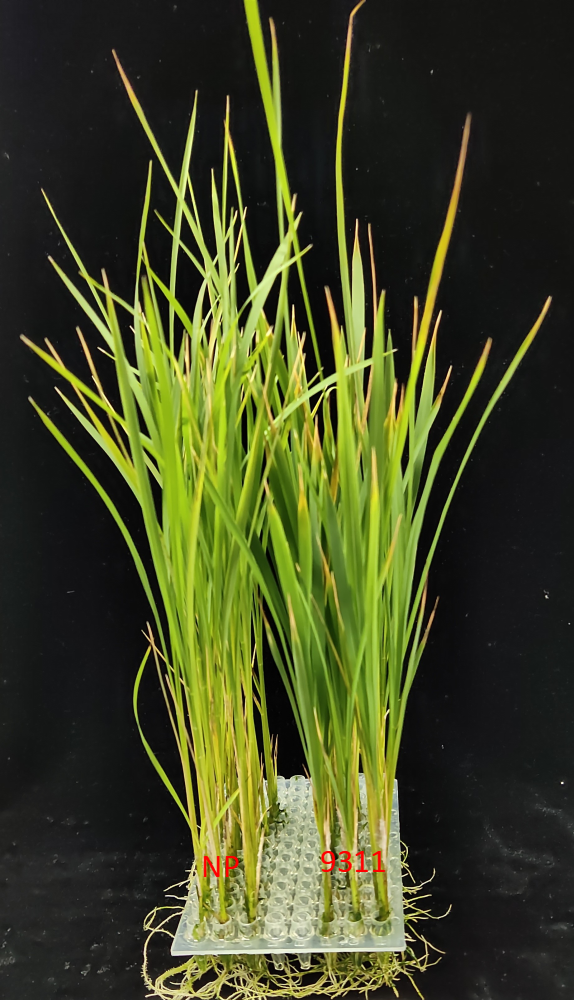

Supplement: S2 Fig — (TIF) [file pone.0284510.s002.tif]

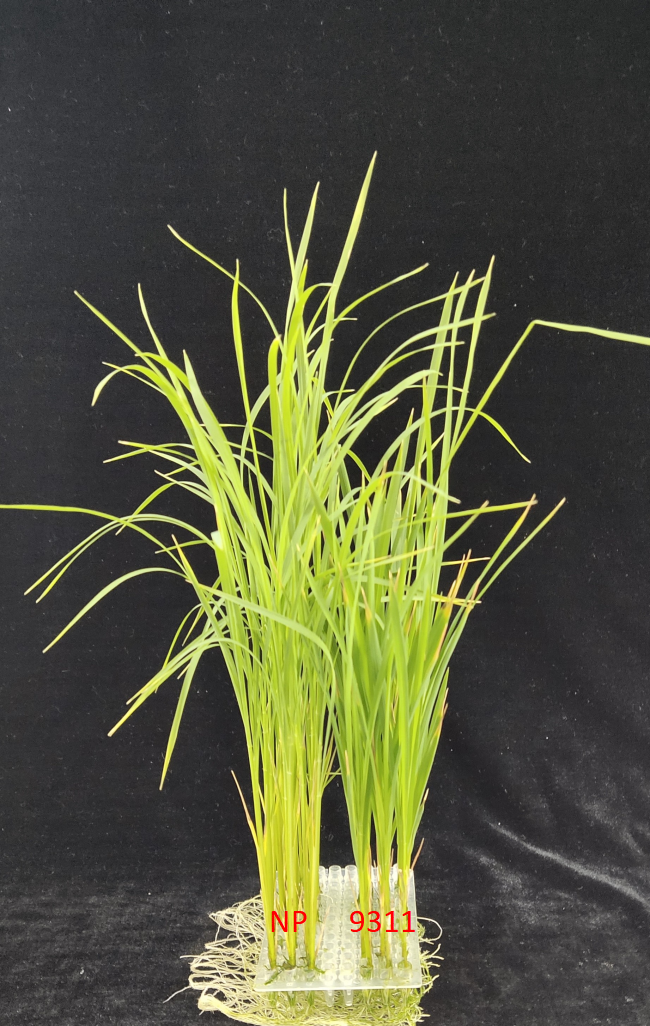

Supplement: S3 Fig — (TIF) [file pone.0284510.s003.tif]

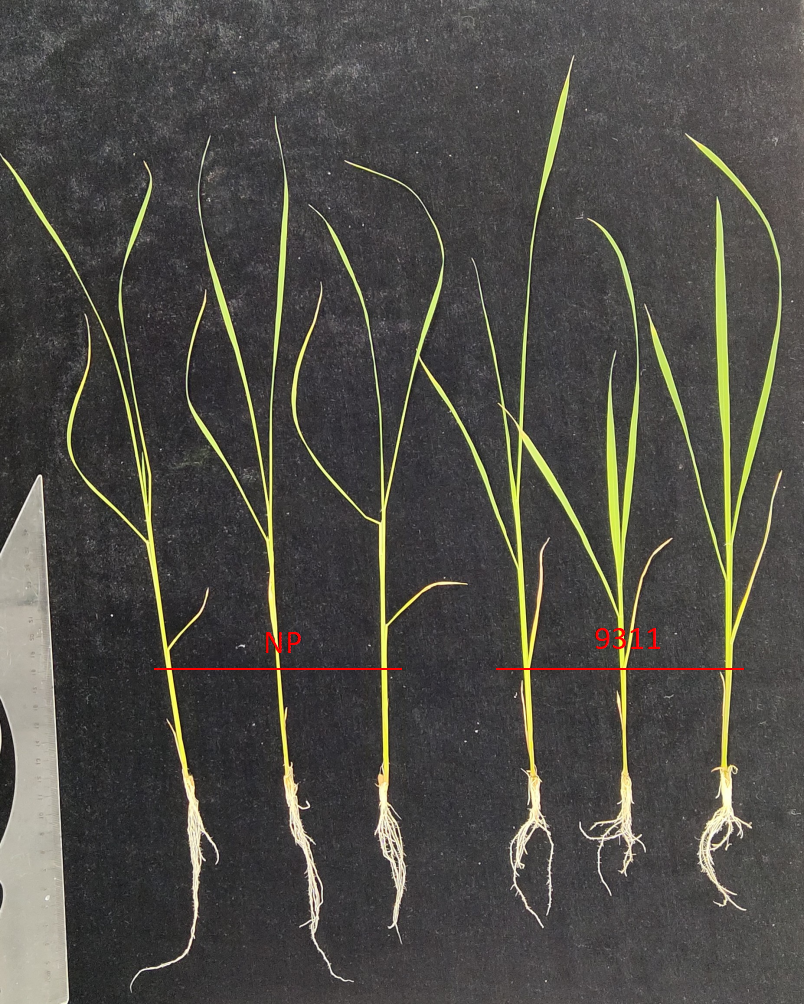

Supplement: S4 Fig — (TIF) [file pone.0284510.s004.tif]

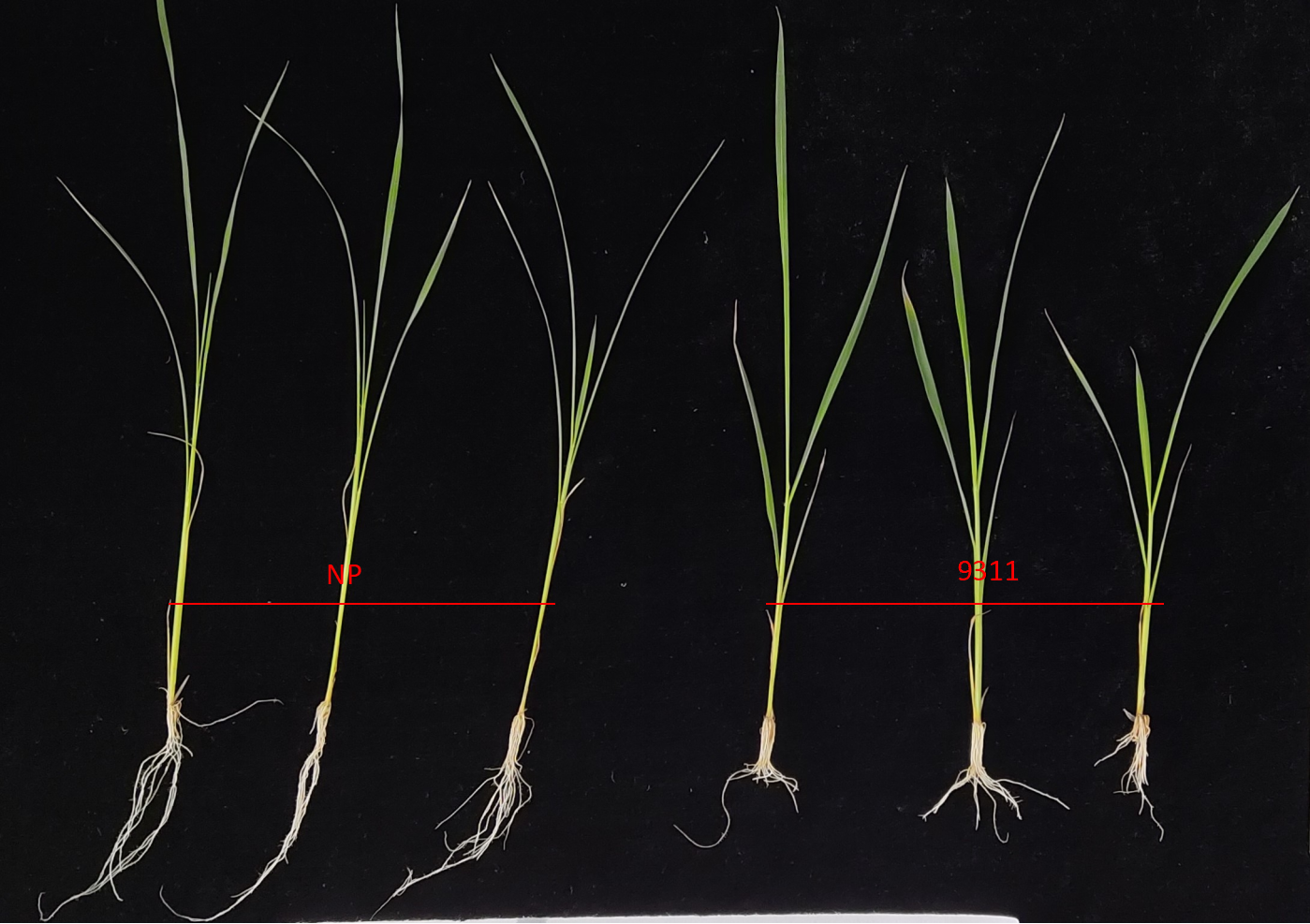

Supplement: S5 Fig — (TIF) [file pone.0284510.s005.tif]
