# Supplementary material for: NP and 9311 are excellent population parents for screening QTLs of potassium-efficient rice
Source: PLoS One. 2023 Apr 14;18(4):e0284510. doi: 10.1371/journal.pone.0284510 (PMC10104323; doi:10.1371/journal.pone.0284510)
Supplement: Supplementary file 2 [file pone.0284510.s006.odt]

| Supplemental table 1. Plant height of twelve rice varieties in different potassium concentration culture solution. |
| --- |
| variety | plant height(/cm) |
| 60 mg/L K+ | 40 mg/L K+ | 20 mg/L K+ | 10 mg/L K+ | 5 mg/L K+ | 4 mg/L K+ | 3 mg/L K+ | 2 mg/L K+ | 1 mg/L K+ | 0 mg/L K+ |
| Wuyunjing | 30.88 | 29.54 | 27.61 | 28.43 | 25.40 | 25.22 | 22.87 | 23.23 | 20.96 | 20.87 |
| J183(IndicaII) | 34.71 | 31.95 | 29.84 | 27.58 | 26.37 | 20.05 | 20.56 | 18.97 | 19.06 | 17.02 |
| 02428 | 38.02 | 37.46 | 36.28 | 34.59 | 27.47 | 26.72 | 22.66 | 20.96 | 19.04 | 16.66 |
| TQ | 42.85 | 35.83 | 38.42 | 36.08 | 30.17 | 27.15 | 25.03 | 21.85 | 19.54 | 15.47 |
| NP | 36.94 | 36.77 | 35.86 | 34.31 | 32.02 | 32.47 | 28.11 | 26.04 | 22.39 | 19.11 |
| 9311 | 34.42 | 32.68 | 30.26 | 29.18 | 25.53 | 23.47 | 28.11 | 21.84 | 18.98 | 16.97 |
| TB309 | 37.91 | 37.31 | 35.24 | 32.64 | 27.10 | 30.43 | 26.21 | 26.38 | 21.72 | 18.89 |
| J193(Iindica I) | 44.75 | 43.65 | 38.25 | 31.39 | 34.73 | 34.19 | 28.96 | 25.94 | 21.56 | 19.49 |
| J189(Indica I) | 30.56 | 33.55 | 27.74 | 28.32 | 20.56 | 23.20 | 18.39 | 20.08 | 12.44 | 13.64 |
| J426(Indica II) | 37.19 | 37.49 | 35.75 | 32.39 | 31.99 | 29.38 | 26.40 | 26.31 | 19.79 | 20.43 |
| J221 (Indica I) | 29.73 | 26.11 | 22.67 | 20.50 | 21.53 | 20.81 | 17.35 | 17.23 | 16.70 | 13.80 |
| J216(IndicaII） | 34.97 | 26.11 | 31.68 | 26.77 | 26.20 | 28.70 | 24.00 | 22.21 | 18.12 | 15.64 |
