# Supplementary material for: NP and 9311 are excellent population parents for screening QTLs of potassium-efficient rice
Source: PLoS One. 2023 Apr 14;18(4):e0284510. doi: 10.1371/journal.pone.0284510 (PMC10104323; doi:10.1371/journal.pone.0284510)
Supplement: Supplementary file 3 [file pone.0284510.s007.odt]

| Supplemental table 2. Root length of twelve rice varieties in different potassium concentration culture solution. |
| --- |
| variety | root length(/cm) |
| 60 mg/L K+ | 40 mg/L K+ | 20 mg/L K+ | 10 mg/L K+ | 5 mg/L K+ | 4 mg/L K+ | 3 mg/L K+ | 2 mg/L K+ | 1 mg/L K+ | 0 mg/L K+ |
| Wuyunjing | 11.41 | 13.53 | 8.92 | 13.51 | 14.88 | 12.46 | 8.09 | 10.29 | 10.29 | 8.28 |
| J183(IndicaII) | 7.49 | 7.49 | 5.65 | 9.75 | 11.99 | 6.34 | 4.72 | 5.14 | 5.21 | 6.32 |
| 02428 | 11.77 | 12.38 | 10.72 | 14.92 | 16.22 | 10.87 | 7.35 | 7.81 | 7.79 | 6.82 |
| TQ | 13.65 | 10.64 | 9.47 | 13.19 | 14.74 | 11.29 | 6.88 | 7.74 | 7.00 | 7.01 |
| NP | 9.23 | 13.39 | 8.51 | 11.64 | 14.48 | 12.77 | 8.77 | 10.13 | 8.45 | 7.71 |
| 9311 | 8.74 | 8.22 | 5.23 | 7.77 | 7.48 | 5.26 | 6.09 | 5.23 | 5.06 | 6.14 |
| TB309 | 10.58 | 12.02 | 8.65 | 10.34 | 12.67 | 11.36 | 8.01 | 8.71 | 8.71 | 7.64 |
| J193(Iindica I) | 12.04 | 9.92 | 9.19 | 11.18 | 16.76 | 9.12 | 9.35 | 7.90 | 8.11 | 8.83 |
| J189(Indica I) | 7.68 | 8.21 | 5.57 | 11.30 | 7.15 | 5.79 | 4.15 | 5.58 | 3.54 | 3.74 |
| J426(Indica II) | 6.97 | 8.46 | 6.67 | 9.91 | 12.15 | 6.79 | 6.00 | 6.69 | 4.56 | 6.26 |
| J221 (Indica I) | 7.76 | 6.26 | 4.22 | 5.20 | 6.99 | 5.60 | 4.26 | 4.29 | 4.48 | 4.15 |
| J216(IndicaII） | 10.01 | 8.56 | 6.63 | 10.30 | 10.63 | 8.68 | 5.13 | 5.43 | 4.67 | 4.71 |
