# Supplementary material for: NP and 9311 are excellent population parents for screening QTLs of potassium-efficient rice
Source: PLoS One. 2023 Apr 14;18(4):e0284510. doi: 10.1371/journal.pone.0284510 (PMC10104323; doi:10.1371/journal.pone.0284510)
Supplement: Supplementary file 4 [file pone.0284510.s008.odt]

| Supplemental table 3. Root number of twelve rice varieties in different potassium concentration culture solution. |
| --- |
| variety | root number |
| 60 mg/L K+ | 40 mg/L K+ | 20 mg/L K+ | 10 mg/L K+ | 5 mg/L K+ | 4 mg/L K+ | 3 mg/L K+ | 2 mg/L K+ | 1 mg/L K+ | 0 mg/L K+ |  |
| Wuyunjing | 15.17 | 16.06 | 15.56 | 16.72 | 14.89 | 11.78 | 13.72 | 10.83 | 10.50 | 7.94 |  |
| J183(IndicaII) | 17.83 | 16.94 | 15.22 | 14.93 | 17.78 | 13.72 | 15.44 | 12.00 | 12.78 | 10.28 |  |
| 02428 | 14.17 | 14.33 | 14.61 | 15.72 | 13.77 | 12.06 | 11.06 | 10.06 | 9.72 | 7.61 |  |
| TQ | 21.00 | 13.28 | 11.61 | 14.17 | 11.39 | 10.78 | 10.35 | 9.11 | 9.17 | 7.44 |  |
| NP | 17.78 | 19.61 | 18.00 | 19.33 | 17.11 | 16.03 | 12.61 | 11.11 | 10.06 | 7.11 |  |
| 9311 | 21.92 | 21.50 | 19.83 | 23.56 | 21.31 | 20.06 | 18.72 | 16.38 | 16.00 | 11.17 |  |
| TB309 | 14.93 | 16.25 | 16.11 | 16.33 | 13.76 | 13.67 | 11.24 | 13.06 | 10.44 | 8.41 |  |
| J193(Iindica I) | 16.87 | 16.44 | 18.44 | 17.67 | 18.35 | 15.27 | 14.96 | 13.22 | 10.61 | 6.67 |  |
| J189(Indica I) | 14.25 | 17.48 | 14.33 | 19.16 | 11.70 | 12.26 | 10.27 | 9.64 | 6.44 | 6.83 |  |
| J426(Indica II) | 15.42 | 16.44 | 20.00 | 19.67 | 19.82 | 17.11 | 17.84 | 14.22 | 11.28 | 11.22 |  |
| J221 (Indica I) | 17.00 | 15.06 | 10.78 | 11.27 | 11.27 | 10.00 | 8.77 | 8.50 | 8.28 | 8.06 |  |
| J216(IndicaII） | 17.94 | 19.83 | 19.25 | 16.67 | 16.38 | 18.80 | 14.04 | 15.39 | 11.39 | 8.50 |  |
