# Supplementary material for: NP and 9311 are excellent population parents for screening QTLs of potassium-efficient rice
Source: PLoS One. 2023 Apr 14;18(4):e0284510. doi: 10.1371/journal.pone.0284510 (PMC10104323; doi:10.1371/journal.pone.0284510)
Supplement: Supplementary file 5 [file pone.0284510.s009.odt]

| Supplemental table 4. Fresh root weight of twelve rice varieties in different potassium concentration culture solution. |
| --- |
| variety | fresh root weight(/g) |
| 60 mg/L K+ | 40 mg/L K+ | 20 mg/L K+ | 10 mg/L K+ | 5 mg/L K+ | 4 mg/L K+ | 3 mg/L K+ | 2 mg/L K+ | 1 mg/L K+ | 0 mg/L K+ |
| Wuyunjing | 0.0841 | 0.1153 | 0.0731 | 0.1339 | 0.1104 | 0.0731 | 0.0550 | 0.0624 | 0.0644 | 0.0362 |
| J183(IndicaII) | 0.0860 | 0.0830 | 0.0501 | 0.0742 | 0.0810 | 0.0564 | 0.0447 | 0.0424 | 0.0517 | 0.0301 |
| 02428 | 0.0844 | 0.0839 | 0.0808 | 0.1188 | 0.0826 | 0.0600 | 0.0365 | 0.0473 | 0.0493 | 0.0268 |
| TQ | 0.0848 | 0.0581 | 0.0518 | 0.0938 | 0.0557 | 0.0490 | 0.0377 | 0.0420 | 0.0450 | 0.0271 |
| NP | 0.0769 | 0.0994 | 0.0634 | 0.1114 | 0.1091 | 0.0691 | 0.0480 | 0.0605 | 0.0567 | 0.0301 |
| 9311 | 0.1373 | 0.1170 | 0.0812 | 0.1359 | 0.0945 | 0.0771 | 0.0694 | 0.0755 | 0.0684 | 0.0538 |
| TB309 | 0.0701 | 0.0750 | 0.0596 | 0.0835 | 0.0687 | 0.0643 | 0.0382 | 0.0598 | 0.0477 | 0.0258 |
| J193(Iindica I) | 0.1273 | 0.1057 | 0.1053 | 0.1196 | 0.1147 | 0.0753 | 0.0595 | 0.0732 | 0.0551 | 0.0286 |
| J189(Indica I) | 0.0579 | 0.0751 | 0.0567 | 0.1260 | 0.0553 | 0.0496 | 0.0358 | 0.0455 | 0.0234 | 0.0208 |
| J426(Indica II) | 0.0956 | 0.1090 | 0.1017 | 0.1324 | 0.1328 | 0.0804 | 0.0630 | 0.0693 | 0.0432 | 0.0313 |
| J221 (Indica I) | 0.1114 | 0.0693 | 0.0424 | 0.0438 | 0.0492 | 0.0457 | 0.0339 | 0.0352 | 0.0361 | 0.0242 |
| J216(IndicaII） | 0.1219 | 0.1192 | 0.0910 | 0.1219 | 0.0492 | 0.1030 | 0.0548 | 0.0712 | 0.0525 | 0.0299 |
