# Supplementary material for: NP and 9311 are excellent population parents for screening QTLs of potassium-efficient rice
Source: PLoS One. 2023 Apr 14;18(4):e0284510. doi: 10.1371/journal.pone.0284510 (PMC10104323; doi:10.1371/journal.pone.0284510)
Supplement: Supplementary file 6 [file pone.0284510.s010.odt]

| Supplemental table 5. Fresh sheath weight of twelve rice varieties in different potassium concentration culture solution. |
| --- |
| variety | fresh sheath weight(/g) |
| 60 mg/L K+ | 40 mg/L K+ | 20 mg/L K+ | 10 mg/L K+ | 5 mg/L K+ | 4 mg/L K+ | 3 mg/L K+ | 2 mg/L K+ | 1 mg/L K+ | 0 mg/L K+ |
| Wuyunjing | 0.1860 | 0.2362 | 0.1851 | 0.1953 | 0.1609 | 0.1569 | 0.1214 | 0.1387 | 0.1190 | 0.0665 |
| J183(IndicaII) | 0.1495 | 0.1452 | 0.1142 | 0.0949 | 0.0981 | 0.0610 | 0.0651 | 0.0635 | 0.0670 | 0.0380 |
| 02428 | 0.2275 | 0.2647 | 0.2775 | 0.1848 | 0.1325 | 0.1146 | 0.0655 | 0.0662 | 0.0605 | 0.0368 |
| TQ | 0.2095 | 0.2010 | 0.2168 | 0.1667 | 0.1102 | 0.1119 | 0.0661 | 0.0629 | 0.0598 | 0.0358 |
| NP | 0.1668 | 0.2210 | 0.1873 | 0.1883 | 0.1696 | 0.1702 | 0.1116 | 0.1076 | 0.0968 | 0.0540 |
| 9311 | 0.1534 | 0.2473 | 0.1532 | 0.1856 | 0.1172 | 0.1061 | 0.0929 | 0.1022 | 0.0874 | 0.0570 |
| TB309 | 0.1624 | 0.1902 | 0.1863 | 0.1544 | 0.1357 | 0.1447 | 0.0843 | 0.1071 | 0.0938 | 0.0576 |
| J193(Iindica I) | 0.2574 | 0.2499 | 0.2407 | 0.1601 | 0.1455 | 0.1372 | 0.0951 | 0.1005 | 0.0653 | 0.0389 |
| J189(Indica I) | 0.1061 | 0.1744 | 0.1209 | 0.1619 | 0.0873 | 0.0845 | 0.0617 | 0.0896 | 0.0404 | 0.0360 |
| J426(Indica II) | 0.1980 | 0.2226 | 0.2234 | 0.1753 | 0.1820 | 0.1336 | 0.1062 | 0.1090 | 0.0857 | 0.0522 |
| J221 (Indica I) | 0.1783 | 0.1252 | 0.1080 | 0.0828 | 0.0849 | 0.0882 | 0.0597 | 0.0659 | 0.0666 | 0.0460 |
| J216(IndicaII） | 0.1890 | 0.2127 | 0.2263 | 0.1875 | 0.1439 | 0.1839 | 0.0844 | 0.0998 | 0.0810 | 0.0437 |
