# Supplementary material for: NP and 9311 are excellent population parents for screening QTLs of potassium-efficient rice
Source: PLoS One. 2023 Apr 14;18(4):e0284510. doi: 10.1371/journal.pone.0284510 (PMC10104323; doi:10.1371/journal.pone.0284510)
Supplement: Supplementary file 7 [file pone.0284510.s011.odt]

| Supplemental table 6. Fresh blade weight of twelve rice varieties in different potassium concentration culture solution. |
| --- |
| variety | fresh blade weight(/g) |
| 60 mg/L K+ | 40 mg/L K+ | 20 mg/L K+ | 10 mg/L K+ | 5 mg/L K+ | 4 mg/L K+ | 3 mg/L K+ | 2 mg/L K+ | 1 mg/L K+ | 0 mg/L K+ |
| Wuyunjing | 0.1222 | 0.1394 | 0.1096 | 0.1353 | 0.1220 | 0.1177 | 0.0958 | 0.0984 | 0.0979 | 0.0640 |
| J183(IndicaII) | 0.1280 | 0.0998 | 0.0882 | 0.0979 | 0.1086 | 0.0736 | 0.0687 | 0.0771 | 0.0792 | 0.0457 |
| 02428 | 0.1538 | 0.1525 | 0.1539 | 0.1317 | 0.1051 | 0.1054 | 0.0685 | 0.0688 | 0.0576 | 0.0335 |
| TQ | 0.1260 | 0.1010 | 0.1364 | 0.1250 | 0.0856 | 0.0961 | 0.0669 | 0.0624 | 0.0598 | 0.0311 |
| NP | 0.0984 | 0.1291 | 0.1024 | 0.1280 | 0.1212 | 0.1168 | 0.0864 | 0.0855 | 0.0804 | 0.0494 |
| 9311 | 0.1271 | 0.1966 | 0.1298 | 0.1530 | 0.1129 | 0.1085 | 0.1040 | 0.1049 | 0.0907 | 0.0633 |
| TB309 | 0.1290 | 0.1117 | 0.1071 | 0.1017 | 0.1016 | 0.1106 | 0.0720 | 0.0872 | 0.0729 | 0.0546 |
| J193(Iindica I) | 0.1592 | 0.1428 | 0.1416 | 0.1128 | 0.1067 | 0.1018 | 0.0885 | 0.0932 | 0.0726 | 0.0387 |
| J189(Indica I) | 0.0815 | 0.1103 | 0.0899 | 0.1178 | 0.0718 | 0.0786 | 0.0597 | 0.0808 | 0.0458 | 0.0374 |
| J426(Indica II) | 0.1320 | 0.1397 | 0.1407 | 0.1400 | 0.1473 | 0.1237 | 0.0961 | 0.1074 | 0.0852 | 0.0581 |
| J221 (Indica I) | 0.1257 | 0.0826 | 0.0579 | 0.0507 | 0.0682 | 0.0779 | 0.0538 | 0.0647 | 0.0657 | 0.0433 |
| J216(IndicaII） | 0.1336 | 0.1534 | 0.1407 | 0.1331 | 0.1133 | 0.1506 | 0.0859 | 0.0977 | 0.0694 | 0.0498 |
