# Supplementary material for: NP and 9311 are excellent population parents for screening QTLs of potassium-efficient rice
Source: PLoS One. 2023 Apr 14;18(4):e0284510. doi: 10.1371/journal.pone.0284510 (PMC10104323; doi:10.1371/journal.pone.0284510)
Supplement: Supplementary file 8 [file pone.0284510.s012.odt]

| Supplemental table 7. Potassium content of twelve rice varieties in 1 mg/L potassium culture solution. |
| --- |
| rice variety | dry plant weight（/g) | plant potassium content（g/100 g) | dry root weight（/g) | potassium content in root（g/100g) | dry sheath weight（/g) | potassium content in sheath（g/100g) | dry blade weight（/g) | potassium content in blade（g/100g) |
| Wuyunjing | 0.0370 | 0.5080 | 0.0079 | 0.4058 | 0.0115 | 0.7549 | 0.0177 | 0.3937 |
| J183(IndicaII) | 0.0293 | 0.5066 | 0.0062 | 0.4389 | 0.0073 | 0.6687 | 0.0158 | 0.4583 |
| 02428 | 0.0248 | 0.5287 | 0.0060 | 0.4540 | 0.0076 | 0.6991 | 0.0113 | 0.4545 |
| TQ | 0.0242 | 0.6570 | 0.0056 | 0.5481 | 0.0074 | 0.8384 | 0.0112 | 0.5917 |
| NP | 0.0302 | 0.4984 | 0.0063 | 0.3619 | 0.0091 | 0.7191 | 0.0149 | 0.4214 |
| 9311 | 0.0361 | 0.5040 | 0.0087 | 0.3670 | 0.0094 | 0.6310 | 0.0180 | 0.5041 |
| TB309 | 0.0308 | 0.4621 | 0.0061 | 0.3235 | 0.0099 | 0.6246 | 0.0148 | 0.4103 |
| J193(Iindica I) | 0.0402 | 0.4987 | 0.0068 | 0.4800 | 0.0113 | 0.7489 | 0.0221 | 0.3764 |
| J189(Indica I) | 0.0149 | 0.4834 | 0.0034 | 0.3636 | 0.0040 | 0.6944 | 0.0074 | 0.4246 |
| J426(Indica II) | 0.0317 | 0.3889 | 0.0059 | 0.3915 | 0.0120 | 0.4457 | 0.0139 | 0.3388 |
| J221 (Indica I) | 0.0242 | 0.5201 | 0.0047 | 0.3777 | 0.0068 | 0.6664 | 0.0127 | 0.4934 |
| J216(IndicaII） | 0.0354 | 0.4257 | 0.0073 | 0.2890 | 0.0098 | 0.5727 | 0.0183 | 0.4011 |
