# Supplementary material for: NP and 9311 are excellent population parents for screening QTLs of potassium-efficient rice
Source: PLoS One. 2023 Apr 14;18(4):e0284510. doi: 10.1371/journal.pone.0284510 (PMC10104323; doi:10.1371/journal.pone.0284510)
Supplement: Supplementary file 9 [file pone.0284510.s013.odt]

| Supplemental table 8. Potassium content of twelve rice varieties in 4 mg/L potassium culture solution. |
| --- |
| rice variety | dry plant weight（/g) | plant potassium content（g/100 g) | dry root weight（/g) | potassium content in root（g/100g) | dry sheath weight（/g) | potassium content in sheath（g/100g) | dry blade weight（/g) | potassium content in blade（g/100g) |
| Wuyunjing | 0.0458 | 0.9401 | 0.0092 | 0.4530 | 0.0143 | 1.5144 | 0.0223 | 0.7728 |
| J183(IndicaII) | 0.0292 | 0.8574 | 0.0070 | 0.4150 | 0.0066 | 1.1565 | 0.0155 | 0.9296 |
| 02428 | 0.0442 | 0.8102 | 0.0083 | 0.5088 | 0.0140 | 1.1765 | 0.0218 | 0.6895 |
| TQ | 0.0424 | 0.9888 | 0.0073 | 0.5820 | 0.0141 | 1.4949 | 0.0210 | 0.7908 |
| NP | 0.0230 | 0.9829 | 0.0089 | 0.4475 | 0.0161 | 1.4801 | 0.0230 | 0.8430 |
| 9311 | 0.0408 | 0.9600 | 0.0094 | 0.4658 | 0.0105 | 1.3693 | 0.0209 | 0.9779 |
| TB309 | 0.0467 | 0.8722 | 0.0085 | 0.4603 | 0.0151 | 1.1980 | 0.0232 | 0.8103 |
| J193(Iindica I) | 0.0478 | 0.8418 | 0.0104 | 0.5041 | 0.0148 | 1.2293 | 0.0226 | 0.7438 |
| J189(Indica I) | 0.0295 | 0.9889 | 0.0067 | 0.6079 | 0.0081 | 1.3192 | 0.0146 | 0.9798 |
| J426(Indica II) | 0.0490 | 0.9971 | 0.0102 | 0.4920 | 0.0137 | 1.5367 | 0.0251 | 0.9057 |
| J221 (Indica I) | 0.0291 | 0.9444 | 0.0063 | 0.4442 | 0.0079 | 1.3422 | 0.0149 | 0.9449 |
| J216(IndicaII） | 0.0555 | 0.9382 | 0.0124 | 0.5484 | 0.0156 | 1.0743 | 0.0275 | 1.0359 |
