# Supplementary material for: NP and 9311 are excellent population parents for screening QTLs of potassium-efficient rice
Source: PLoS One. 2023 Apr 14;18(4):e0284510. doi: 10.1371/journal.pone.0284510 (PMC10104323; doi:10.1371/journal.pone.0284510)
Supplement: Supplementary file 10 [file pone.0284510.s014.odt]

| Supplemental table 9. Potassium content of twelve rice varieties in 40 mg/L potassium culture solution. |
| --- |
| rice variety | dry plant weight（/g) | plant potassium content（g/100 g) | dry root weight（/g) | potassium content in root（g/100g) | dry sheath weight（/g) | potassium content in sheath（g/100g) | dry blade weight（/g) | potassium content in blade（g/100g) |
| Wuyunjing | 0.0635 | 4.3443 | 0.0126 | 3.1142 | 0.0143 | 6.3967 | 0.0280 | 3.2264 |
| J183(IndicaII) | 0.0489 | 4.1807 | 0.0109 | 3.3441 | 0.0140 | 6.0175 | 0.0241 | 3.3441 |
| 02428 | 0.0703 | 4.5110 | 0.0111 | 2.9822 | 0.0283 | 6.0660 | 0.0310 | 3.6347 |
| TQ | 0.0484 | 4.5282 | 0.0078 | 2.9638 | 0.0191 | 6.1278 | 0.0215 | 3.6712 |
| NP | 0.0529 | 4.4528 | 0.0100 | 2.4800 | 0.0190 | 6.6947 | 0.0239 | 3.4902 |
| 9311 | 0.0635 | 4.5749 | 0.0126 | 2.7793 | 0.0228 | 5.6922 | 0.0280 | 4.4726 |
| TB309 | 0.0492 | 4.3163 | 0.0088 | 2.8979 | 0.0181 | 6.1161 | 0.0222 | 3.4089 |
| J193(Iindica I) | 0.0639 | 4.0291 | 0.0125 | 2.8643 | 0.0231 | 5.5956 | 0.0283 | 3.2663 |
| J189(Indica I) | 0.0501 | 4.2736 | 0.0094 | 2.8588 | 0.0160 | 6.2090 | 0.0231 | 3.6054 |
| J426(Indica II) | 0.0635 | 4.1948 | 0.0138 | 2.9346 | 0.0206 | 6.0592 | 0.0291 | 3.4672 |
| J221 (Indica I) | 0.0366 | 3.9310 | 0.0094 | 2.4742 | 0.0108 | 6.1000 | 0.0165 | 3.3423 |
| J216(IndicaII） | 0.0708 | 3.8866 | 0.0161 | 2.9605 | 0.0204 | 5.6451 | 0.0343 | 3.2732 |
