# Supplementary material for: NP and 9311 are excellent population parents for screening QTLs of potassium-efficient rice
Source: PLoS One. 2023 Apr 14;18(4):e0284510. doi: 10.1371/journal.pone.0284510 (PMC10104323; doi:10.1371/journal.pone.0284510)
Supplement: Attachment — Submitted filename: Response to Reviewers.doc [file pone.0284510.s015.doc]

Dear Editor:

We do appreciate the time and effort you and the reviewers dedicated to providing feedback on our manuscript and are grateful for the insightful comments on our paper. We have tried to address all the raised concerns accordingly. All changes are highlighted. Here is a point-by-point response for all comments and concerns.

Reviewer #1

This manuscript by Liu et al. tested twelve high-yielding rice varieties for K efficiency under hydroponic conditions. The NP and 9311 were identified as tolerant and sensitive varieties in low potassium medium and recommended as potential parents for QTL screening based on their contrasting features. The paper consists of adequate originality, scientific quality, relevance to the field of this journal and presentation; therefore, it is suitable to publish after minor revision. My suggestion is as follows:

Question 1: In the title of the manuscript, the authors mentioned NP and 9311 are excellent population parents for screening QTLs of K-efficient rice. Since I haven't seen any breakthrough in the QTL analysis, so I was very excited when I read the paper; however, I did not find any outcome regarding the QTLs identification. The recommendation was based only on the contrasting features of the NP and 9311 under variable K concentration.

Response: Population parents with significant phenotypic differences under low potassium levels were screened from 12 high-yield rice varieties in East Asia in this study, and a scientific and reliable method for phenotypic screening was established, which could publish as a phased result. As we know, exploring new genes in plant potassium-efficient and analyzing its mechanism are hot topics in current research, and different research teams are very competitive in this field. Based on the parents and screening methods established in our lab, we use NP as the donor parent and 9311 as the recipient parent to construct the introversion line population, and we will use the established method to identify the low potassium tolerance of the progeny lines. As the selected parents are rare combinations, it is very promising to locate the QTLs sites different from those of previous generations. We hope that later research results will be published in the journal.

Question 2: In the plant materials section, the authors may incorporate some background details of the used varieties, such as origin, year of release, recommended soil type and zone for different varieties.

Response: We have rewritten the Plant materials section and added the information about the 12 rice varieties used in this paper， such as origin, year of release, recommended soil type and zone for different varieties as marked with blue color. Related to this, we add the following references as marked with green color:

3034. Gui-mao Y. A new medium rice variety Teqing No. 1 with high yield and disease resistance was successfully planted. Technology in Brief 1995:33. <https://doi.org/10.14088/j.cnki.issn0439.8114>

35.	Shicheng L, Shao-kai M. Rice varieties and their pedigrees in China. Shanghai Science and Technology Press. 1991;(24).

36.	Xiaonian W, Qiuping Y, Changming X, Chuanyong W, Linli Z. Breeding and cultivation techniques of a new two-line hybrid early rice combination 'Lingliangyou 193'. South China Agriculture. 2015;9(6):1-2. <https://doi.org/10.19415/j.cnki.1673-890x>

Question 3:  Check the spelling “lever” or “level” in the abstract.

response: We sincerely thank the reviewer for careful reading. As suggested by the reviewer, we have corrected the “lever” into “level” in revised version of the manuscript as marked with red color.

Question 4: Some references are missing or irregular in the text. In table 1, the author cited Ghomi et al. (2013) and YAO et al. (2005) but not indexed in the reference section. Likewise, the citation of the reference Ming-zhe et al. (2005) (lines 302-304) is missing in the manuscript’s text. Authors must follow the journal format for reference writing and could use the PLOS One template to prepare.

Response: Thank you for pointing this out. We have changed the citation format of YAO et al. (2005) and Ming-zhe et al. (2005) (lines 302-304) as marked with purple color, and added the citation as marked with yellow color:
